# A multicenter retrospective study aiming to identify patients who respond well to adsorptive granulomonocytapheresis in moderately to severely active ulcerative colitis

**DOI:** 10.1038/s41424-018-0037-0

**Published:** 2018-07-06

**Authors:** Takayuki Yamamoto, Takayuki Iida, Kentaro Ikeya, Masaichi Kato, Ai Matsuura, Satoshi Tamura, Ryosuke Takano, Shinya Tani, Satoshi Osawa, Ken Sugimoto, Takahiro Shimoyama, Hiroyuki Hanai

**Affiliations:** 1 Inflammatory Bowel Disease Center, Yokkaichi Hazu Medical Center, Yokkaichi, Japan; 2 Center for Gastroenterology and Inflammatory Bowel Disease Research, Hamamatsu South Hospital, Hamamatsu, Japan; 3 First Department of Medicine, Hamamatsu University School of Medicine, Hamamatsu, Japan; 4 Department of Endoscopic and Photodynamic Medicine, Hamamatsu University School of Medicine, Hamamatsu, Japan

## Abstract

**Objectives:**

Adsorptive granulomonocytapheresis (GMA) with the Adacolumn has been introduced as a non-pharmacologic treatment for ulcerative colitis (UC). However, a subset of patients who might respond well to GMA needs to be targeted. This study was conducted at three IBD centers to determine factors affecting the efficacy of GMA in patients with moderately-to-severely active UC.

**Methods:**

From January 2008 to December 2017, a total of 894 active episodes (first attack or relapse) in 593 patients were treated with GMA. Clinical remission was defined as normal stool frequency and no rectal bleeding. Multiple clinical and laboratory parameters at entry were considered for efficacy assessment.

**Results:**

Clinical remission was achieved during 422 (47%) of the 894 treatment cases. In the multivariate analysis, predictors for favorable response to GMA were age ≤60 years, UC duration <1 year, Mayo endoscopic subscore 2 (vs. 3), steroid naïve UC, and biologic naïve UC. Clinical remission rate was 70% in patients with four of the five factors, 52% in patients with three factors, 46% in patients with two factors, 39% in patients with one factor, and 18% in patients with none of these factors. Overall, the clinical remission rate was significantly higher in patients with a greater number of the five predictors (*P* < 0.0001).

**Conclusions:**

GMA appeared to be effective in steroid naïve and biologic naïve patients with short duration of UC. Elderly patients (>60 years) and those with severe endoscopic activity did not respond well to GMA. Additional, well designed, prospective, controlled trials should strengthen our findings.

## Introduction

Adsorptive granulomonocytapheresis (GMA) with the Adacolumn is a novel non-pharmacologic strategy for treating patients with ulcerative colitis (UC)^1–3^. The Adacolumn is filled with cellulose acetate beads as adsorptive leukocytapheresis carriers that selectively adsorb granulocytes, monocytes/macrophages, a significant fraction of platelets together with a small number of lymphocytes (FcγR and complement receptors bearing leukocytes)^4–7^. The underlying rationale for GMA is that selective removal of the cell populations involved in the induction and perpetuation of intestinal inflammation from the peripheral blood without affecting other cells such as lymphocytes and erythrocytes.

In Japan since April 2000 when GMA was approved as one treatment option for patients with active UC by the Japan Ministry of Health, it has been widely used for patients with UC, and to our knowledge, it is now available in the European Union countries. Multiple studies in Japan^8–12^ and Europe^13–16^ found that GMA was safe and therapeutically effective in patients with active UC. Additional evidence to support a therapeutic benefit from GMA should lead to a reduced need for pharmacologic preparations like corticosteroids, immunosuppressants, and biologicals which are associated with serious adverse side effects as additional morbidities^17–19^. Thus, GMA has been applied as an alternative non-pharmacological option in the management of UC. Given that GMA has not been associated with serious long-term adverse events, its position in the treatment of UC is likely to expand.

Nevertheless, a large scale randomized controlled trial (RCT) conducted in North America failed to show efficacy in the induction of clinical remission or response in patients with moderate to severe UC^20^. The difference in GMA efficacy between this RCT and other studies^8–16^ may be attributed to demographic/disease characteristics, medical histories, and past exposure to pharmacologic preparations. A subset of patients who might or might not respond to GMA has not been fully identified. In clinical practice setting, it is important to know which patients are most likely to respond to GMA to avoid futile use of medical resources or widely introduce this safe treatment and to establish its position in the management of UC. This study was conducted at centers with abundant knowledge and experience in GMA therapy with the aim of determining factors affecting the efficacy of GMA in patients with active UC. To our knowledge, this is one of the largest studies evaluating the efficacy of GMA in patients with active UC.

## Methods

### Patients and study design

This was a multicenter retrospective study conducted at three independent institutes in Japan. All three centers regularly receive a large number of patients with inflammatory bowel disease (IBD), and include Yokkaichi Hazu Medical Center, Hamamatsu South Hospital, and Hamamatsu University School of Medicine. The inclusion criteria were: (1) endoscopic and histologic diagnosis of UC, excluding indeterminate colitis; (2) Mayo score^21^ of ≥6 (moderately [scores 6–9] or severely [scores 10–12] active UC); (3) Mayo endoscopic subscore^21^ of 2 (moderate) or 3 (severe); (4) active disease despite receiving one or more of the following medications, 5-aminosalicylic acid (5-ASA) preparations (sulphasalazine, mesalazine), corticosteroids, immunosuppressant (azathioprine, 6-mercaptopurine, tacrolimus, cyclosporine) or biologics (infliximab, adalimumab, golimumab). Alternatively, patients who had not received the above medications due to intolerance or lack of response were eligible. Exclusion criteria were inadequate data available for the analysis in this retrospective study. Inadequate data included lack of demographic, clinical presentation, UC course, history of medical treatment, or an incomplete assessment of disease activity during GMA therapy. In our centers, patients with leukocyte count of <2000/mm^3^, serious infection, serious concomitant cerebral, pulmonary, cardiac, hepatic or renal disorders, bleeding complication, or a history of hypersensitivity reaction to an anticoagulant and patients with megacolon or fulminating UC were not treated with GMA.

### GMA therapy

Each patient received five GMA sessions with the Adacolumn. The frequency (1 to 5/week) of GMA was determined mainly based on the severity of UC. One GMA session was 60 to 120 min at a blood flow rate of 30 mL/min. Session time was also determined according to the severity of disease and patient’s tolerance. Essentially, patients who had clinical improvement after five GMA sessions, but did not achieve clinical remission were given five or six additional GMA sessions. Therefore, the maximum number of GMA sessions applied was 11 sessions during a single GMA treatment course.

Patients receiving 5-ASA preparations, immunosuppressants, or biologics at entry could continue with these medications at the same dose and frequency, but addition of a new medication for UC was not allowed during GMA therapy. Patients who worsened or remained unchanged were not given additional GMA sessions. Instead, they could receive corticosteroids, immunosuppressants, biologics, or surgery if necessary. However, patients who were on corticosteroids at entry, the steroid dose was to be tapered or discontinued in line with clinical improvement during GMA therapy.

### Assessment of clinical efficacy and safety

Clinical assessment was regularly made during the treatment. Adverse events, stool frequency, consistency, presence or absence of abdominal discomfort, tenesmus, rectal bleeding, and mucus discharge were recorded. Clinical laboratory values included differential leukocyte count, hemoglobin, platelet count, C-reactive protein (CRP), total protein, albumin, creatinine, urea, sodium, potassium, chloride, alanine aminotransferase, aspartate aminotransferase, alkaline phosphatase, lactic dehydrogenase, total bilirubin, and blood cholesterol.

The clinical sections of the Mayo score were compared at entry (within 1 week before the first GMA session) and after treatment (within 2 weeks after the last GMA session). Clinical remission was defined as a score of 0 in the clinical section (stool frequency and rectal bleeding) of the Mayo score (normal stool frequency and no rectal bleeding). Clinical improvement (no remission) was defined as a decrease in stool frequency and/or rectal bleeding scores by at least one point.

### Factors affecting clinical response to GMA

As potential factors affecting clinical response to GMA therapy, the following parameters at entry in each patient were evaluated: age, gender, duration of UC before entry, number of prior relapses, duration of the current exacerbation before GMA, severity of UC, endoscopic severity, extent of UC, extra-intestinal manifestations, medications for the current exacerbation (5-ASA, corticosteroids, immunosuppressants, biologic agents), adverse events related to GMA, and laboratory biomarkers at entry (leukocyte, granulocyte, lymphocyte, hemoglobin, platelet, CRP, albumin).

### Endoscopic assessment

At entry, endoscopic evaluation was made in all patients to determine the extent of UC and the most severely affected segment. After treatment, our observations included the most severely affected segment at entry. Then, after the treatment, the most severely inflamed segment was compared relative to baseline. Endoscopic remission, which meant mucosal healing (MH) was defined as a Mayo endoscopic subscore^21^ of 0 or 1 after treatment.

### Statistical analysis

Comparisons of frequencies were analyzed by using the chi-square test with Yates’ correction. Continuous data are presented as the mean ± SE values. The mean values between two groups were compared by using the unpaired *t*-test. The change in data with time was analyzed by the paired *t*-test. To identify factors affecting the efficacy (clinical remission) of GMA, both univariate (chi-square test) and multivariate (multiple regression) analyses were done. *P* < 0.05 was considered significant.

### Ethical considerations

As stated above, in Japan, GMA with the Adacolumn is an officially approved treatment option for patients with IBD. Nonetheless, prior to initiating this investigation, our study protocol was reviewed and approved by the Institutional Review Board at the three study sites.

## Results

### The overall number of GMA sessions

**Fig. 1:**
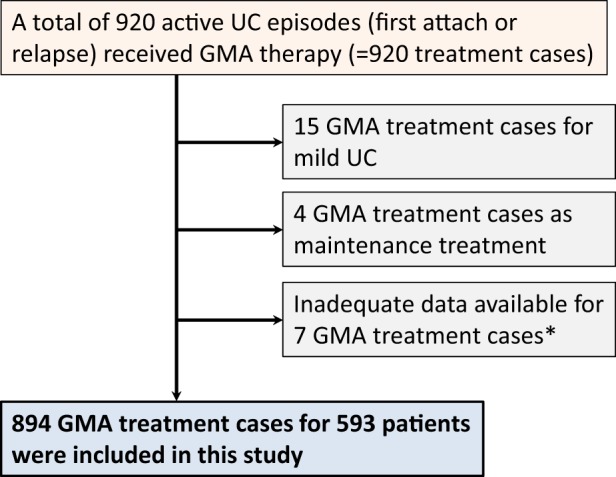
A flow diagram summarizing patient selection and exclusion. *Lack of clinical data like disease duration, number of prior relapses, duration of the current exacerbation, and/or history of medical treatment (four cases) and incomplete assessment of clinical disease activity after GMA therapy (three cases)

During a decade, January 2008–December 2017, a total of 920 UC relapses including first episode cases were treated with GMA at our IBD centers. Twenty-six treatment cases were excluded, and the remaining 894 treatment cases in 593 patients were included for analyses in this study (Fig. 1). Further, during the study period, 374 patients received a single GMA treatment session (374 treatment cases), 154 patients received two treatment sessions (308 treatment cases), 53 patients received three treatment sessions (159 treatment cases), eight patients received four treatment sessions (32 treatment cases), three patients received five treatment sessions (15 treatment cases), and one patient received six treatment sessions (six treatment cases). The demographic characteristics, disease presentation, and medical treatment at entry for the 894 treatment cases are presented in Table 1.

**Table 1:** Demographic characteristics, disease presentation and medical treatment at entry in the 894 GMA treatment cases

Age (mean ± SE)	42 ± 0.5 years
Male: female (*n*)	494: 400
Duration of UC before entry (mean ± SE)	77 ± 4.5 months
Number of prior relapses (mean ± SE)	2.4 ± 0.8
Duration of the current exacerbation before entry (mean ± SE)	3.2 ± 0.05 weeks
Corticosteroids for the current exacerbation (*n*)	711
Cumulative dose of PSL administered before entry (*n*)	
0 g: >0 g, ≤5 g:>5 g	180:601:112
Dose of PSL at entry (mean ± SE)	22.2 ± 0.5 mg/day
Medications for the current exacerbation (*n*)	
5-aminosalicylic acids (Sulfasalazine: mesalazine)	34:810
Thiopurines (Azathioprine: 6-mercaptopurine)	143:15
Calcineurin inhibitors (Tacrolimus: cyclosporine)	7:0
Biologics (Infliximab: adalimumab)	31:36
Disease severity (Mayo score) (*n*)	
Moderate (6–9): severe (10–12)	726:166
Endoscopic severity (Mayo endoscopic subscore) (*n*)	
Moderate (2): Severe (3)	678:216
Extraintestinal manifestations (*n*)	
Arthritis: pyoderma gangrenosum: PSC: others	34:17:3:7
Extent of disease (*n*)	
Proctosigmoiditis: left-sided colitis^a^: pancolitis	113:582:198

*PSL* prednisolone, *PSC* primary sclerosing cholangitis

^a^ Extending to the splenic flexure.

**Fig. 2:**
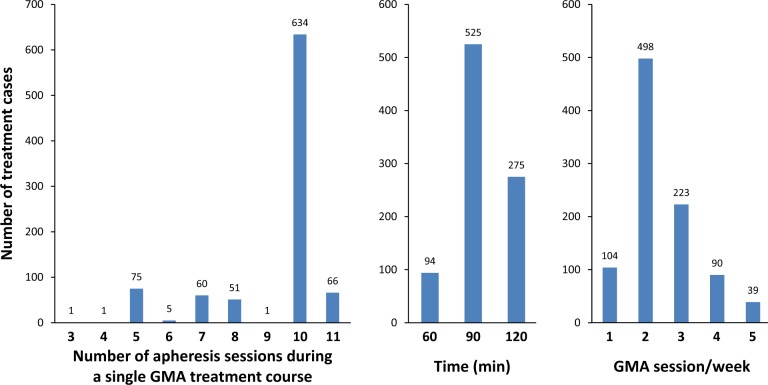
**The number, treatment time, and frequency of apheresis sessions during the 894 GMA treatment cases**

The number of apheresis sessions given during a single GMA treatment course, session time, and the frequency of apheresis sessions are summarized in Fig. 2. As mentioned above, the frequency (1 to 5 sessions/week), treatment time (60–120 min), and the total number of GMA sessions (up to 11) were determined based on the severity of UC, patient tolerance, and response to therapy. The impact of the frequency, time, and the number of GMA sessions on the clinical efficacy could not be evaluated because the outcomes were affected mainly by disease severity and not by the factors related to GMA therapy.

During 24 (3%) of the 894 GMA treatment cases, rapid worsening of UC symptoms like bloody stool ≥20 times/day, high fever (39–40 °C), and acute abdominal discomfort with peritonitis was observed. In these 24 cases, GMA therapy was discontinued, and the patients underwent emergency colectomy. Transient adverse events related to GMA were observed during 290 (32%) of the 894 treatment cases (Table 2). These adverse events were not serious in the majority of patients, but 16 (2%) treatments were discontinued due to severe symptoms (headache and fever five, headache five, nausea three, headache and nausea two, other one). Four GMA treatments were ceased before completing the scheduled protocol because the patients wished to discontinue treatment because of inadequate response, despite reporting no side effects. Therefore, a total of 44 GMA treatments (5%) were ceased in this population because of the need for emergency colectomy, adverse events and patient request for discontinuation.

**Table 2:** Adverse events experienced during the 894 GMA treatment cases

	Number of treatment (%)
Headache	113 (13%)
Fever	74 (8%)
Nausea	54 (6%)
Fatigue	34 (4%)
Dizziness	9 (1%)
Others	6 (0.7%)
**Overall**	**290 (32%)**

### Clinical efficacy

**Fig. 3:**
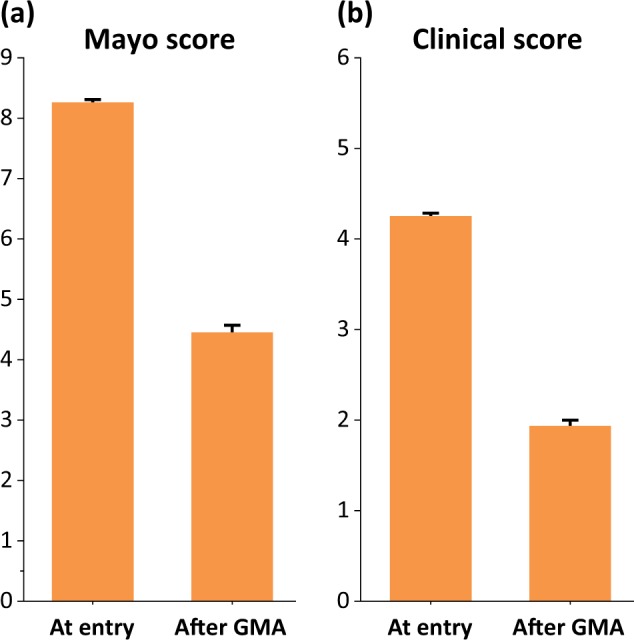
**a** The mean (±SE) Mayo score significantly decreased during GMA treatment (from 8.3 ± 0.05 to 4.5 ± 0.12; *P* < 0.0001). **b** The mean (±SE) score of the clinical section (stool frequency and rectal bleeding, 0–6) also significantly decreased during GMA treatment (from 4.3 ± 0.03 to 1.9 ± 0.07; *P* < 0.0001)

The mean Mayo score significantly decreased during GMA treatment in cases for whom the relevant data for assessment were available after treatment (Fig. 3a). The mean score of the clinical section (stool frequency and rectal bleeding, 0–6) significantly decreased during GMA treatment (Fig. 3b). Clinical remission was achieved during 422 treatment cases (47%), improvement was observed during 227 treatment cases (25%), and no response was observed during 245 treatment cases (27%). During 520 (63%) of 821 GMA treatment cases in which data were available, the dose of prednisolone (PSL) was tapered or discontinued.

### Factors impacting the clinical efficacy of GMA

In univariate analysis, six demographic variables at entry were significantly associated with the likelihood of clinical remission (Table 3). Patients with a short duration of UC (<1 year), first UC episode, steroid naïve as well as biologic naïve patients responded well to GMA. In contrast, elderly patients (>60 years) and those with severe endoscopic activity (Mayo endoscopic subscore 3 vs. 2) did not respond well to GMA. The following factors did not affect the likelihood of clinical remission: Gender, duration of the current exacerbation before GMA, severity and the extent of UC, extra-intestinal manifestations, exposure to 5-ASA preparations, immunosuppressant drugs, and adverse events (Table 3). Laboratory biomarkers at entry (leukocyte, granulocyte, lymphocyte counts, hemoglobin, platelet count, CRP, albumin) were not significantly associated with the clinical remission (Table 4). In multivariate analysis, age, duration of UC, Mayo endoscopic subscore, exposure to steroids, and exposure to biologics were independent significant factors (Table 5).

**Table 3:** The association between clinical parameters at entry of the 894 GMA treatment cases and clinical remission

	Clinical remission rates (%)	*P* ^a^
Age at entry		0.04
<30 years (*n* = 266)	129 (48)	
30–60 years (*n* = 498)	245 (49)	
>60 years (130)	48 (37)	
Gender		0.86
Male (*n* = 494)	235 (48)	
Female (*n* = 400)	187 (47)	
Duration of UC before entry		
<1 year (*n* = 172)	108 (63)	<0.0001
1–5 years (*n* = 564)	249 (44)	
>5 years (*n* = 158)	65 (41)	
Number of prior relapses		
No (First episode) (*n* = 187)	107 (57)	0.003
1–4 (*n* = 559)	257 (46)	
≥5 (*n* = 147)	58 (39)	
Duration of the current exacerbation before entry		0.12
<4 weeks (*n* = 728)	335 (46)	
≥4 weeks (*n* = 164)	87 (53)	
Disease severity		0.09
Moderate (*n* = 726)	353 (48)	
Severe (*n* = 166)	68 (41)	
Endoscopic severity		0.002
Moderate (*n* = 678)	340 (50)	
Severe (*n* = 216)	82 (38)	
Extraintestinal manifestations		0.73
Presence (*n* = 61)	27 (44)	
Absence (*n* = 833)	395 (47)	
Extent of disease		0.08
Proctosigmoiditis (*n* = 113)	63 (56)	
Left-sided colitis (*n* = 582)	274 (47)	
Pancolitis (*n* = 198)	84 (42)	
5-ASA therapy at entry		0.54
Presence (*n* = 844)	401 (48)	
Absence (*n* = 50)	21 (42)	
Exposure to corticosteroids		<0.0001
Presence (*n* = 713)	312 (44)	
Absence (*n* = 180)	110 (61)	
Exposure to immunosuppressants		0.61
Presence (*n* = 159)	72 (45)	
Absence (*n* = 727)	348 (48)	
Exposure to biologics		0.01
Presence (*n* = 67)	21 (31)	
Absence (*n* = 826)	400 (48)	
Adverse events during GMA		0.82
Presence (*n* = 290)	139 (48)	
Absence (*n* = 604)	283 (47)	

^a^The chi-square test.

**Table 4:** The association between laboratory parameters at entry of the 894 GMA treatment cases and clinical remission

	Remission (*n* = 422)	No remission (*n* = 472)	*P* ^a^
Leukocyte count (/mm^3^)	7735 ± 141	7905 ± 136	0.38
Granulocyte count (/mm^3^)	5783 ± 133	5967 ± 130	0.33
Lymphocyte count (/mm^3^)	1352 ± 24	1400 ± 42	0.33
Hemoglobin (g/dL)	12.4 ± 0.9	12.4 ± 0.8	0.59
Platelet count (/mm^3^)	306,445 ± 4582	305,216 ± 4785	0.85
CRP (mg/dL)	1.0 ± 0.5	1.7 ± 0.7	0.41
Albumin (g/dL)	4.0 ± 0.03	4.0 ± 0.03	0.99

Mean ± SE values are presented.

^a^The unpaired t-test.

**Table 5:** Predictive value of clinical and laboratory parameters for clinical remission

	Odds ratio (95% confidence interval)	*P* ^a^
Age at entry:>60 years	0.63 (0.40–0.99)	0.04
Gender: Male	1.02 (0.77–1.34)	0.91
Duration of UC before entry:<1 year	2.63 (1.03–6.67)	0.04
Number of prior relapses: No (First episode)	1.54 (0.61–3.90)	0.36
Duration of the current exacerbation before entry:<4 weeks	1.23 (0.86–1.76)	0.25
Disease severity: Severe	0.88 (0.51–1.51)	0.64
Endoscopic severity: Severe	0.53 (0.31–0.88)	0.01
Extraintestinal manifestations: Presence	0.95 (0.55–1.64)	0.86
Extent of disease: Proctosigmoiditis	1.29 (0.83–1.99)	0.26
5-ASA therapy at entry: Presence	1.28 (0.68–2.38)	0.44
Exposure to corticosteroids: Presence	0.63 (0.41–0.95)	0.03
Exposure to immunosuppressants: Presence	0.74 (0.49–1.10)	0.14
Exposure to biologics: Presence	0.45 (0.24–0.82)	0.01
Adverse events during GMA: Presence	1.10 (0.82–1.49)	0.54

^a^Multiple regression analysis.

**Fig. 4:**
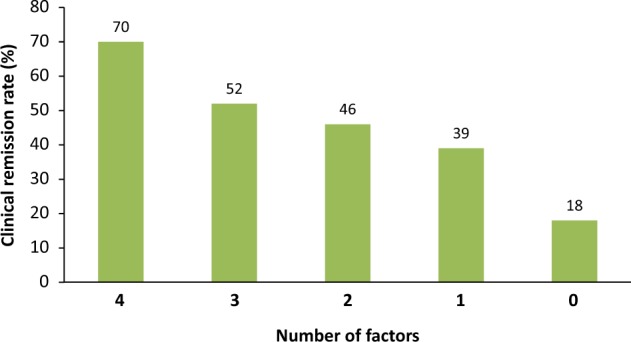
The clinical remission rate was 70% in patients with four of the five predictors for favorable response to GMA (age ≤ 60 years, UC duration < 1 year, Mayo endoscopic subscore 2 [vs. 3], steroid naïve UC, and biologic naïve UC), 52% in patients with three factors, 46% in patients with two factors, 39% in patients with one factor, and 18% in patients with none of these factors. Clinical remission rate was significantly higher in patients with a greater number of these five predictors (*P* < 0.0001)

Based on these findings, predictors for favorable response to GMA in active UC patients were age ≤60 years, UC duration <1 year, Mayo endoscopic subscore 2 (vs. 3), steroid naïve UC, and biologic naïve UC. No patient had all of these five features at entry. Clinical remission rate was 70% in patients with four of these five factors, 52% in patients with three factors, 46% in patients with two factors, 39% in patients with one factor, and 18% in patients with none of these factors. Clinical remission rate was significantly higher in patients with a greater number of these five predictors (Fig. 4).

## Endoscopic evaluation and factors affecting the endoscopic efficacy

**Fig. 5:**
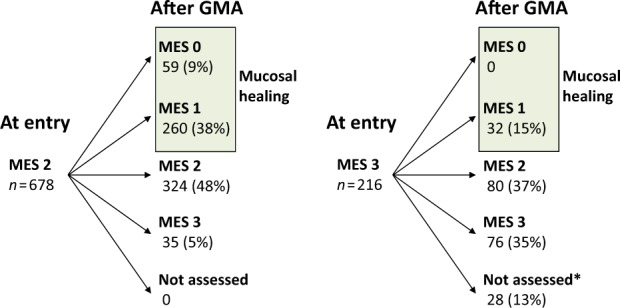
The change in endoscopic severity during the GMA treatment course. Numbers represent treatment cases. MES Mayo endoscopic subscore. *After the treatment, 28 patients could not undergo endoscopic evaluations, 24 required emergency colectomy during GMA therapy and four had serious UC deterioration at the end of the GMA therapy. These 28 patients were listed as non-responders in the endoscopic assessment. Overall, mucosal healing (MH) was observed in 351 of the 894 treatment cases (39%). When sub-grouped, MH was achieved in 378 of 678 treatment cases (47%) with Mayo endoscopic subscore 2 at entry, which was significantly greater than 32 of 216 (15%) with Mayo endoscopic subscore 3 (*P* < 0.0001)

The change in endoscopic severity during the GMA treatment course is presented in Fig. 5. After the treatment, 28 patients could not undergo endoscopic evaluations, 24 required emergency colectomy during GMA therapy and 4 had serious UC deterioration at the end of the GMA therapy. These 28 patients were listed as non-responders in the endoscopic assessment. Overall, MH was observed in 351 of the 894 treatment cases (39%). When sub-grouped, MH was achieved in 378 of 678 treatment cases (47%) with Mayo endoscopic subscore 2 at entry, which was significantly greater than 32 of 216 (15%) with Mayo endoscopic subscore 3 (*P* < 0.0001). Further, MH was achieved in 299 of 422 treatment cases (71%) with clinical remission vs. 52 of 472 (11%) without clinical remission (*P* < 0.0001).

Further, in univariate analysis, six demographic variables at entry were significantly associated with the likelihood of endoscopic remission (MH) seen in the Supplementary Table 1. Patients with a short duration of UC (<1 year), first UC episode and those with proctosigmoiditis, or steroid naïve responded well to GMA endoscopically. In contrast, patients with severe clinical activity (Mayo score 10–12 vs. 6–9) and those with severe endoscopic activity (Mayo endoscopic subscore 3 vs. 2) did not respond well to GMA endoscopically. The following factors did not affect the likelihood of endoscopic remission (MH): Age, gender, duration of the current exacerbation before GMA, extra-intestinal manifestations, exposure to 5-ASA preparations, immunosuppressant, biologic agents, and adverse events (Supplementary Table 1). In multivariate analysis, clinical severity, endoscopic severity, extent of UC, exposure to steroids and exposure to biologics were independent significant factors (Supplementary Table 2).

## Discussion

The efficacy of GMA for active UC has been markedly variable in the past clinical trials^8–16,20^. Many clinicopathological factors appear to affect the efficacy of GMA, including patient demographics, disease characteristics, and past exposure to pharmacologic preparations. Understanding predictive factors of response to GMA is valuable for decision making in therapeutic settings. With this in mind, the present study has several strengths (albeit a retrospective undertaking). We used three large databases from three IBD centers with broad experience and expertise in GMA therapy. We have been treating a large number of patients with GMA in routine clinical practice setting, and accumulated abundant knowledge and experience since 2000 when GMA was first approved in Japan. To our knowledge, this is the largest study with a major focus on identifying predictors of clinical response to GMA. Further, in the previous studies^22–25^, only a single GMA treatment course was used for each patient to evaluate the patient’s response. However, because disease presentation, severity of UC and patient conditions were not identical at each flare-up, the response to GMA was considered to be variable. Therefore, we evaluated the results of all GMA therapies carried out for each patient during the investigation period. Additionally, in this study, many clinical and laboratory parameters were rigorously evaluated by both univariate and multivariate analyses. Thus, this is a large scale study conducted in a practical, real-life setting.

In the present study, predictors of favorable response to GMA were age ≤60 years, UC duration <1 year, Mayo endoscopic subscore 2 (vs. 3), steroid naïve UC, and biologic naïve UC. Clinical remission was achieved in 70% in patients with four of these five factors, 52% in patients with three factors, 46% in patients with two factors, 39% in patients with one factor, and 18% in patients with none of these factors. The rate of clinical remission was significantly higher in patients with a greater number of these five predictors.

In the past, there have been relatively small studies looking for parameters affecting the efficacy of GMA with the Adacolumn in patients with UC. In those studies, the duration of UC before GMA appeared to be an important factor. Suzuki et al.^22^ initially reported a retrospective study, which was aimed at identify predictors of clinical response to GMA. Twenty-eight consecutive patients received up to ten GMA sessions, at two sessions/week. Twenty of 28 patients achieved clinical remission including all eight patients who had their first UC episode. The mean duration of UC in the eight first episode cases was 3.4 months compared with 40.2 months for all 28 patients and 65.4 months for the eight non-responders. They suggested that first UC episode and short disease duration were good predictors of response to GMA and therefore, GMA might be an effective first-line treatmen^22^. Similar findings were reported by Yokoyama et al.^23^ in their multicenter prospective study. Patients with a first UC episode who were drug naïve responded well to GMA and achieved a favorable long-term disease course by avoiding pharmacologic therapy in an early stage of their IBD. In another study, Yokoyama et al.^24^ found that interval between relapse and the first GMA session was an independent significant predictor for clinical response to GMA; the clinical response rate was higher in patients who received GMA immediately after a relapse. Further, the duration of UC before the first GMA session was significantly greater in non-responders as compared with responders. In the present study, a short duration of UC (<1 year) before entry was a predictor of favorable response to GMA. Further, we found that elderly patients (>60 years) showed poor response, which was a new observation in GMA therapy. We believe that additional clinical research is required to ascertain if this is reproducible. However, based on our findings, GMA should be effective for patients with a short history of UC probably because of less exposure to pharmacological preparations.

It appeared to be a significant correlation between the use of steroid before entry and the response to GMA. In a previous study^25^, we found that the dose of PSL administered at entry and the cumulative dose of PSL administered before entry negatively impacted the efficacy of GMA. Yokoyama et al.^24^ also reported that the cumulative dose of PSL before GMA was significantly greater in non-responders than in responders. A number of studies^8,26,27^ evaluated the efficacy of GMA for steroid naïve patients, and reported that it was highly effective (remission rate: 85–88%). Long-term, high dose corticosteroid use potentially produces serious adverse events. If GMA can spare patients from exposure to corticosteroids, the risk of steroid-induced adverse effects should be minimized. This may be of great benefit to patients because severe side effects related to corticosteroids seriously impair health-related quality of life. Our previous investigation found that GMA introduced at an early stage of UC significantly reduces steroid administration and the incidence of steroid-dependency in the long-term^19^. Iida et al.^28^ reported that among patients who responded to GMA, the 3-year sustained clinical remission rates in steroid-naïve, steroid-dependent and steroid-refractory subgroups were 83.3%, 68.8%, and 23.1%, respectively. Steroid-naïve patients appeared to benefit the most from the GMA treatment, and attain a favorable long-term clinical course.

Other studies^29,30^ reported that GMA was effective for less severe IBD, and it was not effective in patients with severely active UC. In this investigation, we found that the full Mayo score (clinical activity) at entry was not a significant predictor, but the endoscopic subscore (endoscopic activity) was a relevant predictor; patients with severe endoscopic inflammation (Mayo endoscopic subscore 3) did not respond well to GMA. From our data, we assumed that endoscopic score was more objective, and directly reflected the response to GMA as compared with the more subjective clinical score.

Endoscopic evaluation during the GMA treatment was also undertaken in this study. Endoscopic remission (MH) was observed in 39% of the treatment cases. MH was more frequently achieved in cases with Mayo endoscopic subscore 2 vs. 3 at entry (47% vs. 15%; statistically significant). Further, the rate of MH was significantly higher in cases with clinical remission vs without clinical remission (71 vs. 11%). Our multivariate analysis showed that clinical severity, endoscopic severity, extent of UC, exposure to steroids or to biologics were independent significant factors. In fact, the predictive factors for endoscopic efficacy were similar to those for clinical efficacy.

Several laboratory biomarkers were found to be associated with GMA efficacy. In one study^24^, GMA was effective in patients with low leukocyte count (remission group 8304.1/μL vs. non-remission group 9572.9/μL). In another study^30^, the erythrocyte sedimentation rate was significantly higher in non-responders than in responders (38.4 mm/hr vs 30.6 mm/hr). In this study, we found that laboratory biomarkers such as total leukocytes, granulocytes, lymphocytes, platelets, hemoglobin, CRP, and albumin were not significantly associated with the clinical efficacy. Right now, we are not sure whether laboratory markers are valuable for the prediction of GMA efficacy. We are now measuring the levels of fecal biomarkers during GMA therapy, and investigating their value for the assessment of disease activity and the prediction of response to the treatment.

This study has certain limitations, which might have impacted our results. First, the methodological design of a retrospective multicenter observational data analyses was undertaken without having been able to influence the GMA treatment protocol. Second, the number of GMA sessions or duration of each session was not fixed. Third, our data were compiled from an open label, clinical practice setting, which means uncontrolled and without a placebo arm. Forth, the follow up time was short. A future, prospective controlled study with a fixed GMA treatment protocol and longer follow-up time should strengthen our findings.

In conclusion, this investigation factoring a large scale patient population treated with GMA in real world therapeutic settings found that 5 demographic features significantly affected the efficacy outcome for GMA. The predictors for favorable response to GMA appeared to be age ≤60 years, UC duration <1 year, Mayo endoscopic subscore 2 (vs 3), steroid naïve, and biologic naïve feature. Accordingly, the clinical remission rate was significantly higher in patients with a greater number of these 5 predictors. Specifically, GMA was more effective in steroid naïve and biologic naïve patients with a short duration of UC. However, in spite of these realities, the Investigation and Research Committee for IBD affiliated to the Japan Ministry of Health has recommended that GMA should be for steroid-refractory or steroid-dependent patients with moderate-to-severe UC. Given that GMA has a very favorable safety profile, has not been associated with any serious long-term adverse event and seems to have steroid sparing effects, its position in the treatment of UC is likely to expand. Nevertheless, since the cost of GMA therapy is relatively expensive, it is important to identify patients who respond well to GMA and avoid futile use of GMA. Additional well designed, prospective, controlled trials should strengthen our findings given that in clinical practice setting, such information can stop futile use of medical resources.

## Study highlights

### What is current knowledge

• Adsorptive GMA with the Adacolumn is a novel non-pharmacologic strategy for treating patients with UC.
• Multiple studies in Japan and Europe found that GMA was safe and therapeutically effective in patients with active UC.
• A subset of patients who might or might not respond to GMA has not been fully identified. In clinical practice setting, it is important to know which patients are most likely to respond to GMA.

### What is new here

• In the present study, predictors of favorable response to GMA were age ≤60 years, UC duration <1 year, Mayo endoscopic subscore 2 (vs. 3), steroid naïve UC, and biologic naïve UC.
• GMA appeared to be effective in steroid naïve and biologic naïve patients with short duration of UC.
• Elderly patients (>60 years) and those with severe endoscopic activity did not respond well to GMA.

## Electronic supplementary material

Supplementary Tables
