# Correction: A multicenter retrospective study aiming to identify patients who respond well to adsorptive granulomonocytapheresis in moderately to severely active ulcerative colitis

**DOI:** 10.1038/s41424-018-0073-9

**Published:** 2018-11-14

**Authors:** Takayuki Yamamoto, Takayuki Iida, Kentaro Ikeya, Masaichi Kato, Ai Matsuura, Satoshi Tamura, Ryosuke Takano, Shinya Tani, Satoshi Osawa, Ken Sugimoto, Takahiro Shimoyama, Hiroyuki Hanai

**Affiliations:** 1 Inflammatory Bowel Disease Center, Yokkaichi Hazu Medical Center, Yokkaichi, Japan; 2 Center for Gastroenterology and Inflammatory Bowel Disease Research, Hamamatsu South Hospital, Hamamatsu, Japan; 3 First Department of Medicine, Hamamatsu University School of Medicine, Hamamatsu, Japan; 4 Department of Endoscopic and Photodynamic Medicine, Hamamatsu University School of Medicine, Hamamatsu, Japan

Correction to: *Clinical and Translational Gastroenterology*;10.1038/s41424-018-0037-0; published online 06 July 2018

This Article was originally published under Nature Research’s License, but has now been made available under a CC BY 4.0 license. The PDF and HTML versions of the article have been modified accordingly.

