# Supplementary material for: A multicenter retrospective study aiming to identify patients who respond well to adsorptive granulomonocytapheresis in moderately to severely active ulcerative colitis
Source: Clin Transl Gastroenterol. 2018 Jul 6;9(7):170. doi: 10.1038/s41424-018-0037-0 (PMC6033883; doi:10.1038/s41424-018-0037-0)
Supplement: Supplementary file 1 — Supplementary Tables [file 41424_2018_37_MOESM1_ESM.docx]

| **Supplementary Table 1** The association between clinical parameters at entry of the 894 GMA treatment cases and endoscopic remission (mucosal healing) | | |
| --- | --- | --- |
|  | Clinical remission rates (%) | P* |
| Age at entry |  | 0.49 |
| <30 years (n=266) | 108 (41) |  |
| 30–60 years (n=498) | 198 (40) |  |
| >60 years (130) | 45 (35) |  |
| Gender |  | 0.19 |
| Male (n=494) | 184 (37) |  |
| Female (n=400) | 167 (42) |  |
| Duration of UC before entry |  |  |
| <1 year (n=172) | 90 (52) | <0.0001 |
| 1–5 years (n=564) | 236 (42) |  |
| >5 years (n=158) | 25 (16) |  |
| Number of prior relapses |  |  |
| No (First episode) (n=187) | 97 (52) | <0.0001 |
| 1–4 (n=559) | 234 (42) |  |
| ≥5 (n=147) | 20 (14) |  |
| Duration of the current exacerbation before entry |  | 0.85 |
| <4 weeks (n=728) | 288 (40) |  |
| ≥4 weeks (n=164) | 63 (38) |  |
| Disease severity |  | <0.0001 |
| Moderate (n=726) | 325 (45) |  |
| Severe (n=166) | 24 (14) |  |
| Endoscopic severity |  | <0.0001 |
| Moderate (n=678) | 319 (47) |  |
| Severe (n=216) | 32 (15) |  |
| Extraintestinal manifestations |  | 0.14 |
| Presence (n=61) | 18 (30) |  |
| Absence (n=833) | 333 (40) |  |
| Extent of disease |  | <0.0001 |
| Proctosigmoiditis (n=113) | 73 (65) |  |
| Left-sided colitis (n=582) | 233 (40) |  |
| Pancolitis (n=198) | 45 (23) |  |
| 5-ASA therapy at entry |  | 0.80 |
| Presence (n=844) | 330 (39) |  |
| Absence (n=50) | 21 (42) |  |
| Exposure to corticosteroids |  | <0.0001 |
| Presence (n=713) | 245 (34) |  |
| Absence (n=180) | 106 (59) |  |
| Exposure to immunosuppressants |  | 0.63 |
| Presence (n=159) | 66 (42) |  |
| Absence (n=727) | 284 (39) |  |
| Exposure to biologics |  | 0.08 |
| Presence (n=67) | 19 (28) |  |
| Absence (n=826) | 331 (40) |  |
| Adverse events during GMA |  | 0.28 |
| Presence (n=290) | 106 (37) |  |
| Absence (n=604) | 245 (41) |  |
| * The chi-square test. | | |

| **Supplementary Table 2** Predictive value of clinical parameters for endoscopic remission (mucosal healing) | | |
| --- | --- | --- |
|  | Odds ratio  (95% confidence interval) | P* |
| Age at entry: >60 years | 0.78 (0.48–1.27) | 0.32 |
| Gender: Male | 0.75 (0.56–1.02) | 0.07 |
| Duration of UC before entry: <1 year | 2.22 (0.79–6.25) | 0.13 |
| Number of prior relapses: No (First episode) | 2.13 (0.47–6.25) | 0.15 |
| Duration of the current exacerbation before entry: <4 weeks | 1.18 (0.79–1.75) | 0.41 |
| Disease severity: Severe | 0.41 (0.21–0.78) | 0.007 |
| Endoscopic severity: Severe | 0.30 (0.16–0.57) | 0.0002 |
| Extraintestinal manifestations: Presence | 0.73 (0.40–1.36) | 0.33 |
| Extent of disease: Proctosigmoiditis | 1.99 (1.26–3.15~~)~~ | 0.003 |
| 5-ASA therapy at entry: Presence | 0.87 (0.44–1.69) | 0.69 |
| Exposure to corticosteroids: Presence | 0.41 (0.27–0.64) | <0.0001 |
| Exposure to immunosuppressants: Presence | 0.52 (0.26–1.01) | 0.053 |
| Exposure to biologics: Presence | 0.51 (0.33–0.80) | 0.003 |
| Adverse events during GMA: Presence | 0.95 (0.68–1.35) | 0.79 |
| * Multiple regression analysis. | | |
